# MiR‐9 is involved in TGF‐β1‐induced lung cancer cell invasion and adhesion by targeting SOX7

**DOI:** 10.1111/jcmm.13120

**Published:** 2017-03-07

**Authors:** Lichun Han, Wei Wang, Wei Ding, Lijian Zhang

**Affiliations:** ^1^ Department of Oncology The Affiliated Hospital of Qingdao University Qingdao Shandong China; ^2^ School of Pharmacy Qingdao University Qingdao Shandong China; ^3^ Department of General Medicine The Affiliated Hospital of Qingdao University Qingdao Shandong China

**Keywords:** microRNA‐9, SOX7, transforming growth factor‐beta 1, lung cancer, invasion, adhesion

## Abstract

MicroRNA (miR)‐9 plays different roles in different cancer types. Here, we investigated the role of miR‐9 in non‐small‐cell lung cancer (NSCLC) cell invasion and adhesion *in vitro* and explored whether miR‐9 was involved in transforming growth factor‐beta 1 (TGF‐β1)‐induced NSCLC cell invasion and adhesion by targeting SOX7. The expression of miR‐9 and SOX7 in human NSCLC tissues and cell lines was examined by reverse transcription‐quantitative polymerase chain reaction. Gain‐of‐function and loss‐of‐function experiments were performed on A549 and HCC827 cells to investigate the effect of miR‐9 and SOX7 on NSCLC cell invasion and adhesion in the presence or absence of TGF‐β1. Transwell–Matrigel assay and cell adhesion assay were used to examine cell invasion and adhesion abilities. Luciferase reporter assay was performed to determine whether SOX7 was a direct target of miR‐9. We found miR‐9 was up‐regulated and SOX7 was down‐regulated in human NSCLC tissues and cell lines. Moreover, SOX7 expression was negatively correlated with miR‐9 expression. miR‐9 knockdown or SOX7 overexpression could suppress TGF‐β1‐induced NSCLC cell invasion and adhesion. miR‐9 directly targets the 3′ untranslated region of SOX7, and SOX7 protein expression was down‐regulated by miR‐9. TGF‐β1 induced miR‐9 expression in NSCLC cells. miR‐9 up‐regulation led to enhanced NSCLC cell invasion and adhesion; however, these effects could be attenuated by SOX7 overexpression. We concluded that miR‐9 expression was negatively correlated with SOX7 expression in human NSCLC. miR‐9 was up‐regulated by TGF‐β1 and contributed to TGF‐β1‐induced NSCLC cell invasion and adhesion by directly targeting SOX7.

## Introduction

Lung cancer is the leading cause of cancer‐related deaths worldwide 1, with the 5‐year survival rate of approximately 16% 2. NSCLC accounts for about 85% of lung cancers, and most of the patients with NSCLC were diagnosed at an advanced stage 3. Therefore, it is necessary to identify novel biomarkers and therapeutic targets for the diagnosis and treatment of this aggressive disease.

MicroRNAs (miRNAs or miRs) are small non‐coding RNAs which negatively regulate gene expression by binding to the 3′ untranslated regions (UTRs) of target mRNAs 4, 5, 6. miRNAs play essential roles in multiple biological processes, such as development, differentiation, metabolism and tumorigenesis 6, 7. Over the past decades, an increasing number of miRNAs have emerged as oncogenes and tumour suppressors in lung cancer pathogenesis 8, 9.

MiR‐9 has various expression patterns in diverse human cancers, and it plays different roles in different cancer types 10, 11, 12, 13. Xu *et al*. found miR‐9 expression is increased in human NSCLC tissues compared with the adjacent noncancerous tissues 14, 15. Up‐regulated miR‐9 expression was correlated with adverse clinical features and unfavourable survival, indicating that miR‐9 is a poor prognosis biomarker in patients with NSCLC. However, the direct role of miR‐9 in the regulation of NSCLC cell invasion and adhesion and its related mechanisms have not yet been fully elucidated.

SRY‐Box 7 (SOX7) is a transcription factor belonging to the SOX family. It is involved in the regulation of various developmental processes 16, 17, 18. Recently, SOX7 has been proposed to play tumour‐suppressive roles in several cancers, such as colon, breast, prostate and liver cancers 19, 20, 21. Li *et al*. 22 found that compared with the matched adjacent normal tissues, SOX7 mRNA expression was significantly down‐regulated in human lung adenocarcinoma tissues. The reduced SOX7 expression was correlated with multiple poor prognostic indicators of patients with lung adenocarcinoma. Thus, SOX7 was suggested to be a novel tumour suppressor in lung cancer 23.

TGF‐β1 is an important cytokine in cancer progression 24, 25. Extensive studies have demonstrated that TGF‐β1 was able to promote invasion and metastasis of various tumour cells 26, 27. In this study, we investigated the role of miR‐9 and SOX7 in TGF‐β1‐induced NSCLC metastasis *in vitro*. Further, we identified whether miR‐9 was involved in TGF‐β1‐induced cell invasion and adhesion by targeting SOX7 in NSCLC. This study may provide a novel molecular mechanism underlying NSCLC metastasis and help for developing new therapeutic strategies for NSCLC.

## Materials and methods

### Tissue samples

This study has been approved by the Ethics Committee of The Affiliated Hospital of Qingdao University. Written informed consent was obtained from all patients before enrolment, and this research was carried out according to the World Medical Association Declaration of Helsinki. Samples were collected from 30 NSCLC tissues and 30 matched adjacent tissues. Among these patients, 16 were male and 14 were female, with the median age of 52 years.

### Cell culture and transfection

The A549, HCC827 and NCI‐H1299 cell lines were obtained from the Cell Bank, Chinese Academy of Sciences (Shanghai, China), and the HEK293, HBE, SK‐LU‐1 and NCI‐H460 cell lines were purchased from the American Type Culture Collection (ATCC; Manassas, VA, USA). The cells were cultured in Dulbecco's modified Eagle medium (DMEM; Invitrogen, Carlsbad, CA, USA) containing 10% foetal bovine serum (FBS; Invitrogen) and maintained at 37°C in an atmosphere of 5% CO_2_. TGF‐β1 (PeproTech, Rocky Hill, NJ, USA) was used to treat the cells at the concentration of 5 ng/ml for 24 hrs. Cell transfection was performed using Lipofectamine 2000 (Invitrogen) according to the manufacturer's instructions. The miR‐negative control (NC) (5′‐UUGUACU ACACAAAA GUACUG‐3′), miR‐9 mimic (sense: 5′‐UCUUUGGUUAUCUAGCUGUAUGA‐3′; antisense: 5′‐AUACAGCUAGAUAACCAAAGAUU‐3′) and miR‐9 inhibitor (antisense: 5′‐UCAUAC AGCUAGAUAACCAAAGA‐3′) were synthesized by Biomics Biotech (Nantong, Jiangsu, China). The pEGFP‐C1 and SOX7‐pEGFP‐C1 constructs were purchased from Shenzhen Zhonghong Boyuan Biological Technology Co., Ltd (Shenzhen, China). miR‐NC, miR‐9 mimic and miR‐9 inhibitor were transfected at a final concentration of 50 nM. pEGFP‐C1 and SOX7‐pEGFP‐C1 plasmids were transfected at a final concentration of 100 nM.

### Luciferase reporter assay

The wild‐type and mutant SOX7 3′ untranslated region (3'UTR) sequences were amplified by polymerase chain reaction and ligated into the pMIRREPORT luciferase vector (Ambion, Austin, TX, USA) to yield pMIR‐SOX7 3′‐UTR (SOX7 3′‐UTR). The HEK293 cells were seeded into the six‐well plates and allowed to reach 60‐80% confluence. The cells were then cotransfected with wild‐type/mutant SOX7 3′‐UTR and miR‐9 mimic/miR‐NC using Lipofectamine 2000. 24 hrs later, the cells were harvested and lysed. Luciferase reporter assay was performed on the Dual‐Luciferase Reporter Assay System (Promega, Madison, WI, USA) according to the manufacturer's instructions. The firefly luciferase activity was normalized to Renilla luciferase activity.

### Reverse transcription–quantitative PCR (RT‐qPCR)

Total RNA was isolated from tissues or cells using the RNeasy/miRNeasy Mini kit (Qiagen, Hilden, Germany).1 μg of total RNAs was reverse‐transcribed to complementary DNAs using the SuperScript II reverse transcriptase (Invitrogen) according to the manufacturer's instructions. Real‐time PCR were performed on a 7500 Real‐Time PCR System (Applied Biosystems, Foster City, CA, USA) using the SYBR Green PCR Master Mix (Applied Biosystems). The following primers were used: miR‐9: 5′‐tctttggttatctagctgtatga‐3′ (sense), 5′‐tggtgtcgtggagtcg‐3′ (antisense); U6: 5′‐ctcgcttcggca gcaca ‐3′ (sense), 5′‐aacgcttcacgaatttgcgt‐3′ (antisense); SOX7: 5′‐cctctccttcttgtgccttg‐3′ (sense), 5′‐gggagacagcaactctcagg‐3′ (antisense); GAPDH: 5′‐cgaccactttgtcaagctca‐3′ (sense), 5′‐aggggagattcagtgtggtg‐3′ (antisense). The threshold cycle (Ct) of target genes was normalized to that of the internal control.

### Western blot

The total protein was extracted from the lung cancer tissues and NSCLC cell lines using the Western and IP cell lysis buffer (Sangon Biotech, Shanghai, China). Protein concentration was quantified using the BCA Protein Assay Kit (Sangon Biotech). Equal amounts of protein were separated by 10% SDS‐PAGE and transferred onto the nitrocellulose membranes (Millipore, Billerica, MA, USA). The membranes were blocked with 5% skim milk in tris‐buffered saline containing 0.1% Tween 20 (TBST) at 4°C overnight. After washing with TBST, the membranes were incubated with primary antibodies against SOX7 (1:500; Santa Cruz Biotechnology, Santa Cruz, CA, USA) and GAPDH (1:2000; Santa Cruz Biotechnology) at 4°C overnight, followed by incubation with horseradish peroxidase‐conjugated secondary antibody (Santa Cruz Biotechnology) for 1 hr at 37°C. GAPDH antibody was used as a normalization control. The proteins were visualized with the SuperSignal West Pico Chemiluminescent Substrate (Pierce, Rockford, IL, USA). The density of protein bands was quantified using the ImageJ software (NIH, Bethesda, MD, USA).

### Cell invasion assay

The transwell inserts with 8‐μm pore polycarbonate membrane (Corning, New York, NY, USA) were pre‐coated with Matrigel (BD Biosciences, Franklin Lakes, NJ, USA). 1 × 10^5^ cells in serum‐free medium were seeded in the upper chamber of the transwell inserts. Medium containing 10% FBS was added to the lower chamber. Following incubation for 24 hrs, the non‐invaded cells on the upper surface of the membrane were removed with a swab cotton. The invaded cells on the lower surface of the membrane were fixed in 95% ethanol, stained with haematoxylin and counted under a light microscope.

### Cell adhesion assay

The 96‐well plates were pre‐coated with fibronectin (Sigma‐Aldrich; Louis, MO, USA) and blocked with 1% bovine serum albumin (BSA; Sigma‐Aldrich) at 37°C for 2 hrs. 3 × 10^4^ cells in serum‐free medium were seeded into the 96‐well plates. Following incubation for 2 hrs, the adhesive cells were fixed in 4% paraformaldehyde and then stained with 0.5% crystal violet (Sangon Biotech). Sodium dodecyl sulphate (Amresco, Solon, OH, USA) was used to dissolve the crystals. The absorbance at 570 nm was measured using a microplate reader.

### Statistical analysis

All statistical analyses were performed using SPSS 19.0 software (IBM SPSS, Armonk, NY, USA). The data were presented as the mean ± S.D. Differences between two groups were analysed using the Student's *t*‐test, and differences between multiple groups were analysed using analysis of variance (anova). Correlations were analysed using Spearman correlation analysis. *P* values less than 0.05 were considered statistically significant.

## Results

### The correlation analysis of miR‐9 and SOX7 expression in human NSCLC tissues

**Figure 1:**
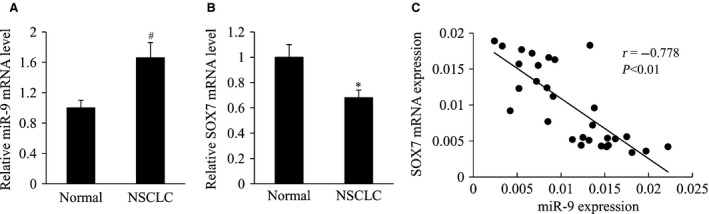
The correlation analysis of miR‐9 and SOX7 expression in human NSCLC tissues. (**A**) Relative miR‐9 mRNA level, (**B**) relative SOX7 mRNA level in human NSCLC and the adjacent normal tissues. (**C**) The correlation of miR‐9 mRNA expression and SOX7 mRNA expression in human NSCLC tissues. *P < 0.05 and ^#^ *P* < 0.01 compared with the normal.

We detected the mRNA expression of miR‐9 and SOX7 by RT‐qPCR in 30 cases of NSCLC tissue samples. We found the expression of miR‐9 was significantly up‐regulated, while the expression of SOX7 was significantly down‐regulated in NSCLC tissues compared with the adjacent normal tissues (Fig. 1A and B). Furthermore, miR‐9 expression was negatively correlated with SOX7 expression in human NSCLC tissues (Fig. 1C).

### The expression of miR‐9 and SOX7 in human NSCLC cell lines

**Figure 2:**
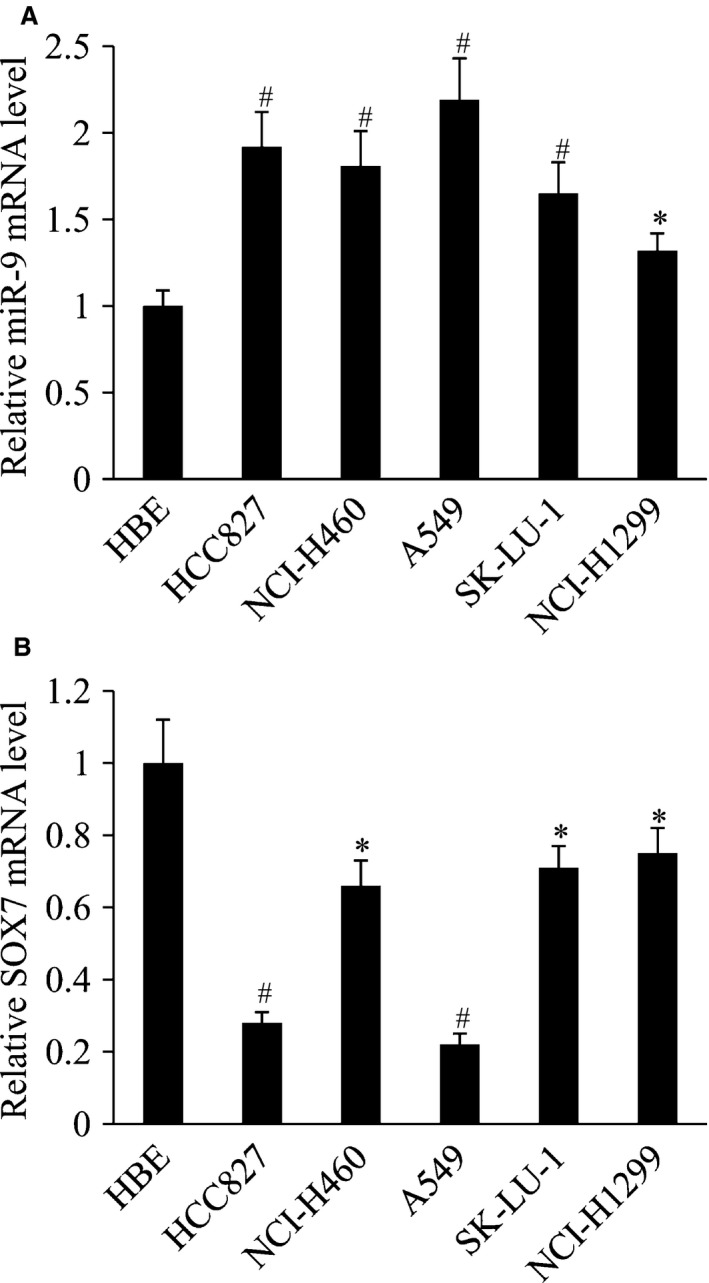
The expression of miR‐9 and SOX7 in human NSCLC cell lines. (**A**) Relative miR‐9 mRNA level, (**B**) relative SOX7 mRNA level in human NSCLC cell lines. *P < 0.05 and ^#^ *P* < 0.01 compared with the HBE cell line.

We detected the mRNA expression of miR‐9 and SOX7 by RT‐qPCR in five NSCLC cell lines (HCC827, NCI‐H460, A549, SK‐LU‐1 and NCI‐H1299). The normal human bronchial epithelial (HBE) cell line was used as a control. The results showed miR‐9 expression was inversely correlated with SOX7 expression in human NSCLC cell lines. miR‐9 expression was significantly up‐regulated, while SOX7 expression was significantly down‐regulated in NSCLC cell lines compared with those in HBE cell line (Fig. 2).

### MiR‐9 knockdown suppressed TGF‐β1‐induced NSCLC cell invasion and adhesion

**Figure 3:**
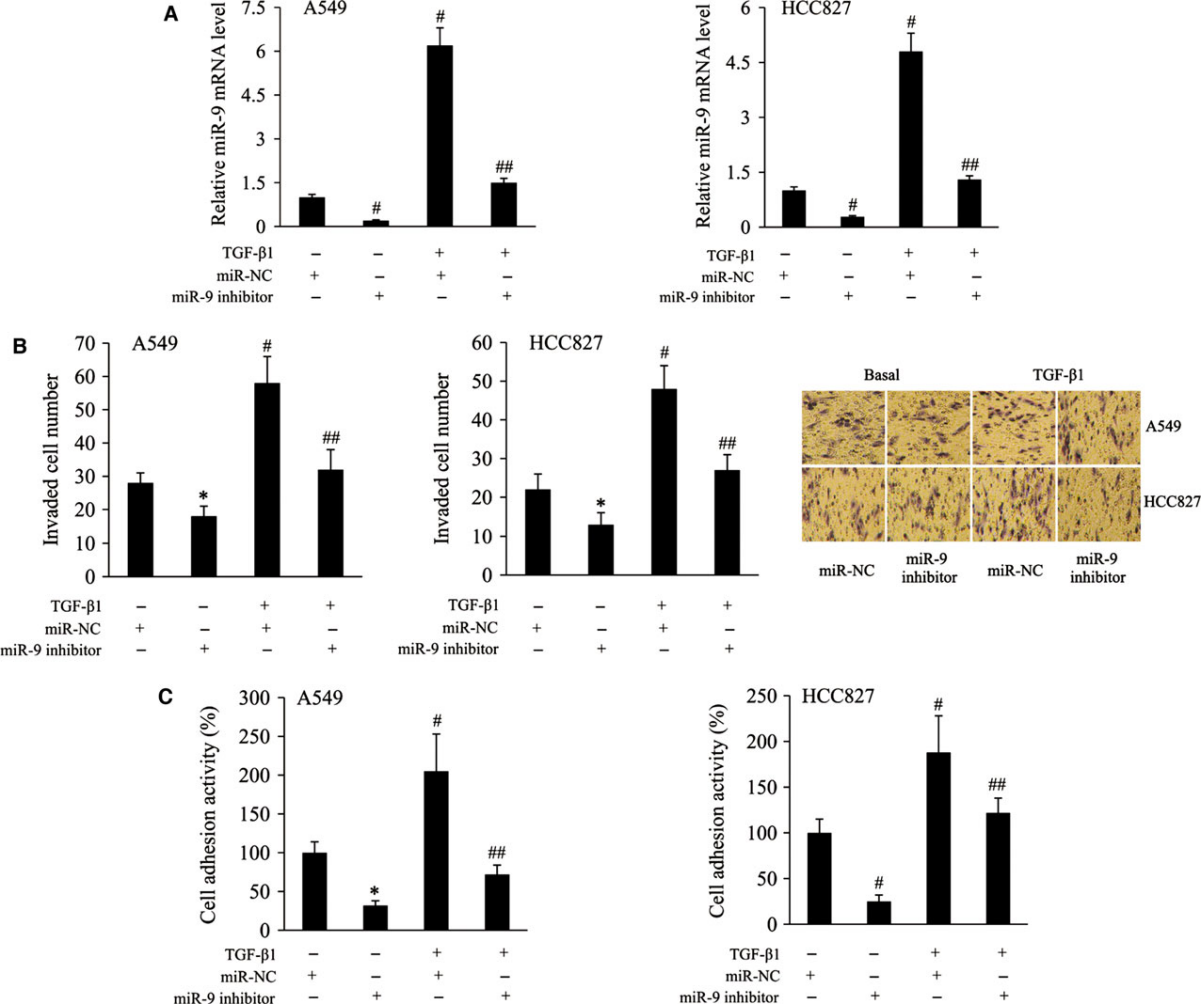
miR‐9 knockdown suppressed TGF‐β1‐induced NSCLC cell invasion and adhesion. (**A**) Relative miR‐9 mRNA level in, (**B**) invaded cell number of, (**C**) cell adhesion activity of A549 and HCC827 cells following transfection with the miR‐NC/miR‐9 in the absence or presence of TGF‐β1. *P < 0.05 and ^#^ *P* < 0.01 compared with the miR‐NC; ^##^ *P* < 0.01 compared with the TGF‐β1+miR‐NC.

To investigate the effect of miR‐9 on TGF‐β1‐induced NSCLC cell invasion and adhesion, miR‐9 inhibitor was transfected into the A549 and HCC827 cells, both of which have high endogenous miR‐9 expression. As demonstrated by RT‐qPCR analysis, miR‐9 expression level in miR‐9 inhibitor‐transfected cells was reduced to 20‐30% of the miR‐NC‐transfected cells in both the A549 and HCC827 cells with or without TGF‐β1 treatment (Fig. 3A). Cell invasion and cell adhesion assays revealed that TGF‐β1 treatment significantly increased cell invasion and adhesion activities of A549 and HCC827 cells; however, the invaded cell number and cell adhesion activity were significantly reduced with miR‐9 knockdown in both the A549 and HCC827 cells with or without TGF‐β1 treatment (Fig. 3B and C).

### SOX7 overexpression suppressed TGF‐β1‐induced NSCLC cell invasion and adhesion

**Figure 4:**
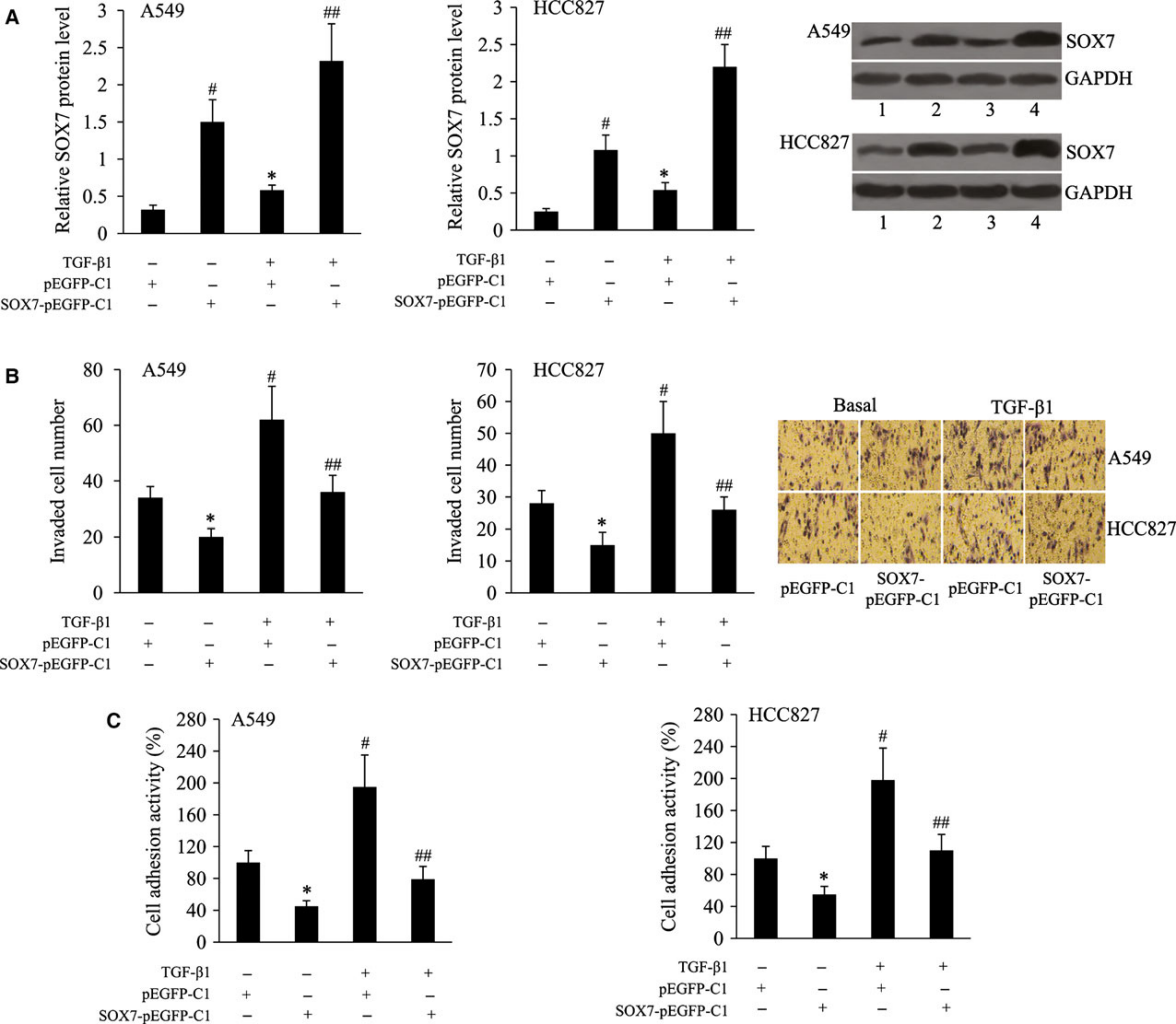
SOX7 overexpression suppressed TGF‐β1‐induced NSCLC cell invasion and adhesion. (**A**) Relative SOX7 protein level in, (**B**) invaded cell number of, (**C**) cell adhesion activity of A549 and HCC827 cells following transfection with the pEGFP‐C1/SOX7‐pEGFP‐C1 in the absence or presence of TGF‐β1. Lane 1, pEGFP‐C1; lane 2, SOX7‐pEGFP‐C1; lane 3, TGF‐β1+pEGFP‐C1; lane 4, TGF‐β1+SOX7‐pEGFP‐C1. *P < 0.05 and ^#^ *P* < 0.01 compared with the pEGFP‐C1; ^##^ *P* < 0.01 compared with the TGF‐β1+ pEGFP‐C1.

To enhance SOX7 expression in A549 and HCC827 cells, SOX7‐pEGFP‐C1 plasmid was transfected into these two cell lines. As demonstrated by Western blot analysis, the expression level of SOX7 protein in SOX7‐pEGFP‐C1‐transfected cells was increased approximately fourfold over the vector control‐transfected cells (Fig. 4A). We found SOX7 overexpression resulted in significantly decreased invaded cell number and cell adhesion activity in both the A549 and HCC827 cells without TGF‐β1 treatment. TGF‐β1‐induced cell invasion and adhesion were also suppressed by SOX7 overexpression (Fig. 4B and C).

### TGF‐β1 induced miR‐9 and SOX7 overexpression in NSCLC cells

**Figure 5:**
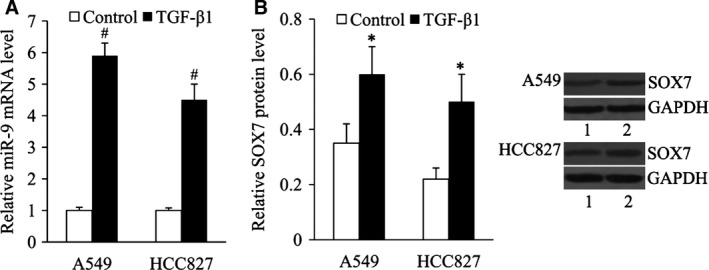
TGF‐β1 induced miR‐9 and SOX7 overexpression in NSCLC cells. (**A**) Relative miR‐9 mRNA level, (**B**) relative SOX7 protein level in A549 and HCC827 cells in the absence or presence of TGF‐β1. Lane 1, control; lane 2, TGF‐β1. *P < 0.05 and ^#^ *P* < 0.01 compared with the control.

We examined the expression of miR‐9 and SOX7 in A549 and HCC827 cells treated with 10 ng/ml TGF‐β1 for 24 hrs. The results of RT‐qPCR showed that miR‐9 expression was significantly increased by sixfold and 4.5‐fold in A549 and HCC827 cells after TGF‐β1 treatment, respectively (Fig. 5A). Western blot results showed that SOX7 expression was significantly increased in A549 and HCC827 cells after TGF‐β1 treatment (Fig. 5B).

### SOX7 was a direct target of miR‐9

**Figure 6:**
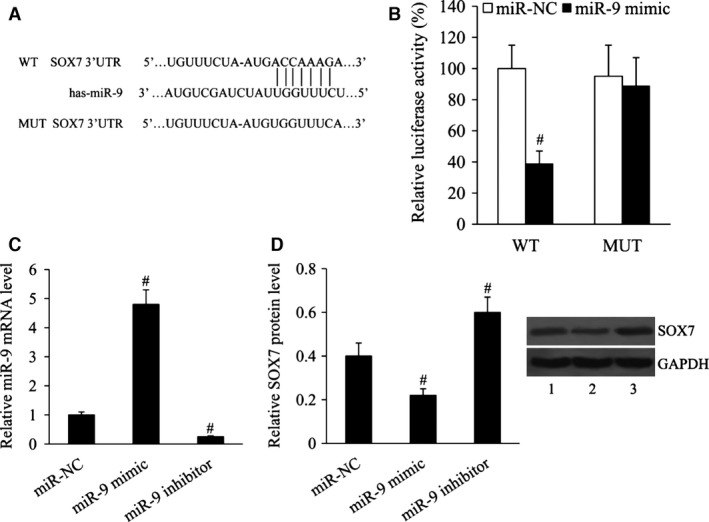
SOX7 was a direct target of miR‐9. (**A**) Bioinformatics predicted the binding site of miR‐9 with SOX7 3′UTR. (**B**) The relative luciferase activity of SOX7 wild‐type or mutant 3′UTR in HEK293 cells following transfection with the miR‐9 mimic. (**C**) Relative miR‐9 mRNA level. (**D**) Relative SOX7 protein level in HEK293 cells following transfection with the miR‐9 mimic or miR‐9 inhibitor. Lane 1, miR‐NC; lane 2, miR‐9 mimic; lane 3, miR‐9 inhibitor. ^#^ *P* < 0.01 compared with the miR‐NC.

To identify whether SOX7 was a miR‐9‐directed target gene, we predicted the interaction site of miR‐9 and SOX7 3′UTR. The predicted binding sequences of wild‐type or mutant SOX7 3′UTR were shown in Figure 6A, and they were transfected into the HEK293 cells together with miR‐9 mimic or miR‐NC. The results from luciferase reporter assay showed that the transcriptional activity of wild‐type SOX7 3′UTR was significantly decreased by miR‐9 mimic. The transcriptional activity of mutant SOX7 3′UTR was not affected by miR‐9 mimic (Fig. 6B). To investigate whether miR‐9 could regulate SOX7 expression, the miR‐9 mimic and miR‐9 inhibitor were transfected into the HEK293 cells. The results of RT‐qPCR analysis demonstrated that miR‐9 was overexpressed in miR‐9 mimic‐transfected cells, and its expression was suppressed in miR‐9 inhibitor‐transfected cells (Fig. 6C). Western blot results showed that the relative protein level of SOX7 was significantly decreased in the cells transfected with the miR‐9 mimic, but increased in the cells transfected with the miR‐9 inhibitor (Fig. 6D).

### SOX7 was involved in miR‐9‐mediated NSCLC cell invasion and adhesion

**Figure 7:**
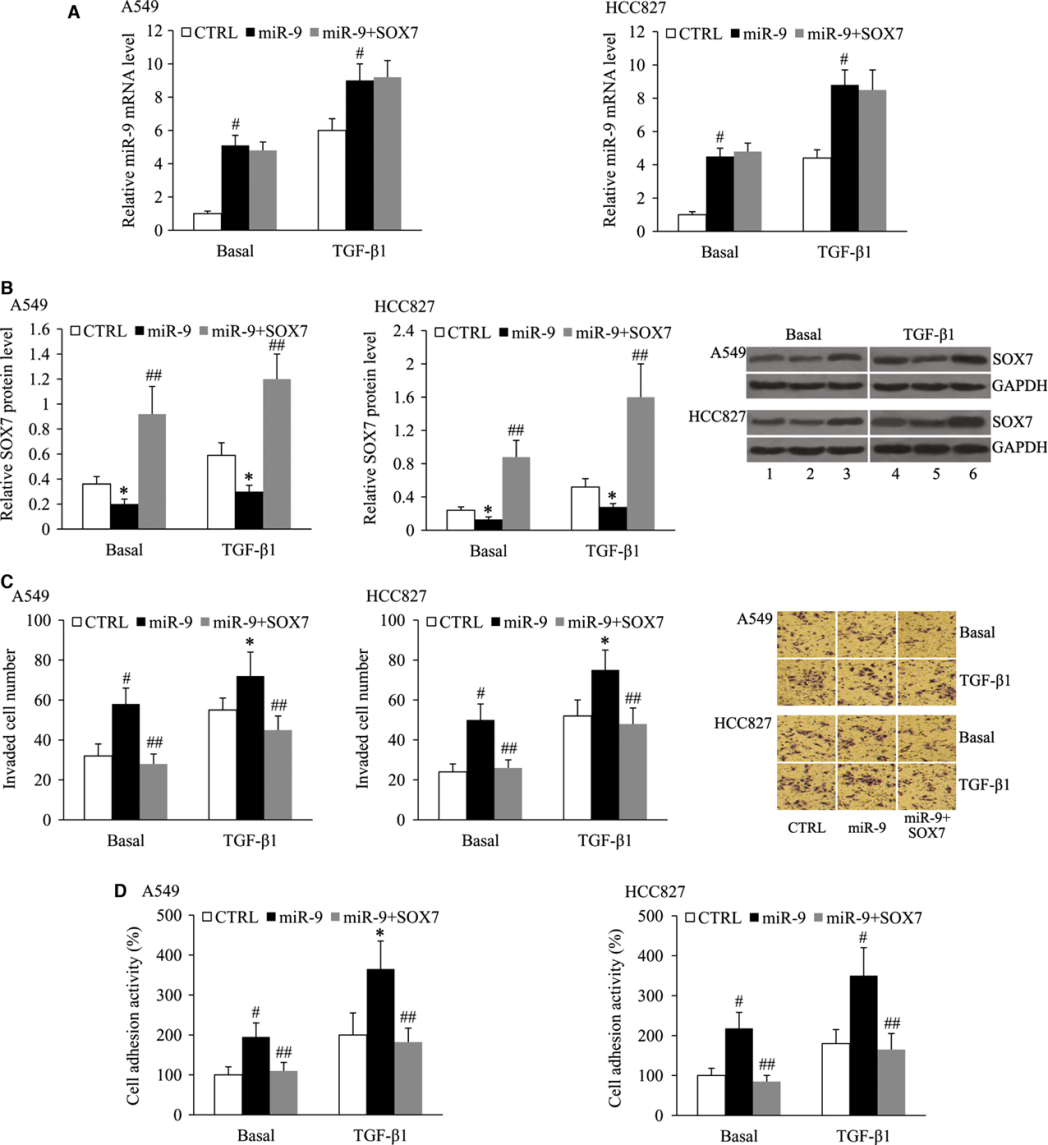
SOX7 is involved in miR‐9‐mediated NSCLC cell invasion and adhesion. (**A**) Relative miR‐9 mRNA level in, (**B**) relative SOX7 protein level in, (**C**) invaded cell number of, (**D**) cell adhesion activity of A549 and HCC827 cells following transfection with the miR‐9 mimic and SOX7‐pEGFP‐C1 in the absence or presence of TGF‐β1. Lane 1, CTRL; lane 2, miR‐9 mimic; lane 3, miR‐9 mimic+SOX7‐pEGFP‐C1; lane 4, TGF‐β1+CTRL; lane 5, TGF‐β1+miR‐9 mimic; lane 6, TGF‐β1+miR‐9 mimic+SOX7‐pEGFP‐C1. *P < 0.05 and ^#^ *P* < 0.01 compared with the CTRL; ^##^ *P* < 0.01 compared with the miR‐9 mimic.

To identify whether SOX7 mediates the effect of miR‐9 on NSCLC cell invasion and adhesion, the A549 and HCC827 cells were transfected with miR‐9 mimic and SOX7‐pEGFP‐C1 plasmid, followed by treating with 10 ng/ml TGF‐β1 for 24 hrs. As demonstrated in Figure 7A, the increased expression of miR‐9 in miR‐9 mimic‐transfected cells was not affected by SOX7 overexpression. The relative protein level of SOX7 was significantly increased in the cells transfected with the miR‐9 mimic and SOX7‐pEGFP‐C1 plasmid compared with the cells transfected with the miR‐9 mimic only (Fig. 7B). The results of cell invasion and cell adhesion assays showed that miR‐9 mimic transfection led to increased invaded cell number and cell adhesion ability of both the A549 and HCC827 cells with or without TGF‐β1 treatment; however, these effects could be attenuated by SOX7 overexpression (Fig. 7C and D).

## Discussion

MiR‐9 has been demonstrated to be involved in tumorigenesis and development 10, 11, 12, 13. A recent study has reported that miR‐9 is up‐regulated and acts as a pro‐metastatic miRNA in breast cancer cells. miR‐9 could down‐regulate the expression of the key metastasis‐suppressing protein E‐cadherin, leading to increased cell motility and invasiveness 28. Contrasting reports were observed in colorectal cancer and hepatocellular carcinoma cells, in which miR‐9 plays a tumour‐suppressive role by suppressing cell migration and invasion 13, 29. These reports indicate miR‐9 acts either as an oncogene or tumour suppressor depending on the cancer types. Consistent with previous studies 14, 15, we found miR‐9 was up‐regulated in human NSCLC tissues and cell lines. Further, this study for the first time showed that miR‐9 knockdown suppressed NSCLC cell invasion and adhesion *in vitro*. TGF‐β1 is an important regulator in NSCLC metastasis 30, 31, 32. In the present study, we found TGF‐β1‐induced cell invasion and adhesion were also inhibited by miR‐9 knockdown. These results confirmed that miR‐9 promotes NSCLC metastasis and it acts as an oncogene in NSCLC.

MiRNAs exert their functions by negatively regulating their target genes 6. According to miRanda (http://www.microrna.org/microrna/home.do), SOX7 was predicted to be a target of miR‐9. SOX7 has been demonstrated to play tumour‐suppressive roles in human tumours 19, 20, 21, 22, 23. SOX7 down‐regulation has been observed in many human cancers, and the decreased SOX7 expression is correlated with poor prognosis of patients with cancer 22, 23, 33. SOX7 mRNA and protein expression were decreased or silenced in the majority of NSCLC cell lines, as well as the NSCLC tissue samples compared with the matched normal lung 23. Forced expression of SOX7 in NSCLC cell lines significantly suppressed cell growth and induced cell apoptosis 23. In this study, SOX7 was found to be down‐regulated in human NSCLC tissues and cell lines. Moreover, SOX7 expression was negatively correlated with miR‐9 expression in clinical tissue samples and cell lines. Numerous functional studies revealed that SOX7 overexpression inhibits cell proliferation and colony formation and induces cell apoptosis. On the contrary, SOX7 knockdown significantly increased cell proliferation, migration and invasion of non‐tumourigenic cells 34, 35, 36. Our *in vitro* experiments demonstrated that SOX7 acts as a tumour suppressor in NSCLC cells and SOX7 overexpression suppressed TGF‐β1‐induced metastasis in NSCLC cells.

Another important finding from this study was that SOX7 was a direct target of miR‐9. The luciferase reporter assay demonstrated that miR‐9 directly targets the 3′UTR of SOX7 and inhibits the transcription activity of SOX7 mRNA. In addition, SOX7 protein expression was regulated by miR‐9. TGF‐β1 has been implicated in the pathogenesis of lung cancers 30, 31, 32. Interestingly, in the present study, we found the expression of miR‐9 and SOX7 was up‐regulated by TGF‐β1 treatment in NSCLC cells, despite the observation that SOX7 was a direct target of miR‐9. Why was SOX7 not decreased by TGF‐β1 treatment? We speculate that although miR‐9 directly targeted SOX7, the expression of SOX7 in the cells could be regulated by many other molecules. Maybe some other molecules which were regulated by TGF‐β1 could promote SOX7 expression. We further found that miR‐9 up‐regulation led to enhanced NSCLC cell invasion and adhesion; however, these effects could be attenuated by SOX7 overexpression. These findings revealed that miR‐9 promotes TGF‐β1‐induced NSCLC metastasis by directly targeting SOX7.

It is well known that a single miRNA can bind to several mRNAs and several miRNAs can bind to a specific mRNA. Previous studies have shown that miR‐9 regulates FoxO1 expression in NSCLC and FoxO1 is the downstream effector of TGF‐β signalling 14, 37, 38; however, the role of miR‐9‐FoxO1 in TGF‐β1‐induced NSCLC cell invasion and adhesion is still unknown. SOX7 was predicted to be the target of miR‐181 according to miRanda. Choi *et al*. reported that miR‐181 is a tumour suppressor in NSCLC and TGF‐β1 was able to up‐regulate miR‐181 39, 40. In the present study, we revealed miR‐9‐SOX7 was involved in TGF‐β1‐induced NSCLC cell invasion and adhesion. What is the relationship between miR‐181, SOX7 and miR‐9? What is the role of miR‐181‐SOX7‐miR‐9 in the TGF‐β1‐induced NSCLC cell invasion and adhesion? These issues need to be explored in the further studies.

In summary, miR‐9 expression was negatively correlated with SOX7 expression in human NSCLC tissue samples and cell lines. miR‐9 was up‐regulated by TGF‐β1 and contributed to TGF‐β1‐induced NSCLC cell invasion and adhesion by directly targeting SOX7. This study provided a novel molecular mechanism underlying NSCLC metastasis and suggested TGF‐β1/miR‐9/SOX7 axis to be a novel therapeutic target in NSCLC.

## Conflict of interest

The authors confirm that there are no conflict of interests.

## Authors’ contributions

LH and WW designed the experiments, WD and LZ performed the experiments, LZ analysed the data, and LH wrote the manuscript. All authors read and gave their approval for the final version of the manuscript.
